# Complementarity of Long‐Reads and Optical Mapping in Parkinson's Disease for Structural Variants

**DOI:** 10.1002/acn3.70332

**Published:** 2026-02-07

**Authors:** André Fienemann, Theresa Lüth, Susen Schaake, Carolin Gabbert, Marius Möller, Hauke Busch, Katja Lohmann, Jonas A. Gustafson, Danny E. Miller, Kensuke Daida, Manabu Funayama, Nobutaka Hattori, Samia Ben Sassi, Faycel Hentati, Matthew J. Farrer, Kristian K. Ullrich, Christine Klein, Joanne Trinh

**Affiliations:** ^1^ Institute of Neurogenetics University of Lübeck Lübeck Germany; ^2^ Institute of Experimental Dermatology University of Lübeck Lübeck Germany; ^3^ Division of Genetic Medicine, Department of Pediatrics University of Washington and Seattle Children's Hospital Seattle Washington USA; ^4^ Molecular and Cellular Biology Program University of Washington Seattle Washington USA; ^5^ Department of Laboratory Medicine and Pathology University of Washington Seattle Washington USA; ^6^ Brotman Baty Institute for Precision Medicine University of Washington Seattle Washington USA; ^7^ Molecular Genetics Section, Laboratory of Neurogenetics National Institute on Aging, National Institutes of Health Bethesda Maryland USA; ^8^ Center for Alzheimer's and Related Dementias (CARD) National Institute on Aging and National Institute of Neurological Disorders and Stroke, National Institutes of Health Bethesda Maryland USA; ^9^ Department of Neurology, Faculty of Medicine Juntendo University Tokyo Japan; ^10^ Research Institute for Diseases of Old Age, Graduate School of Medicine Juntendo University Tokyo Japan; ^11^ Neurodegenerative Disorders Collaborative Laboratory RIKEN Center for Brain Science Wako Japan; ^12^ Department of Neurology National Institute of Neurology Mongi Ben Hmida Tunis Tunisia; ^13^ Faculty of Medicine of Tunis University of Tunis Tunis Tunisia; ^14^ Service de Neurologie Institut National de Neurologie Tunis Tunisia; ^15^ McKnight Brain Institute, Department of Neurology College of Medicine, University of Florida Gainesville Florida USA; ^16^ Division Scientific IT Group Max Planck Institute for Evolutionary Biology Plön Germany

**Keywords:** nanopore long‐read sequencing, optical genome mapping, Parkinson's disease, structural variants

## Abstract

**Objective:**

Long‐read sequencing and optical genome mapping technologies have the ability to detect large and complex structural variants. This has led to the discovery of novel pathogenic variants in neurodegenerative movement disorders. Thus, we aimed to systematically compare the SV detection capabilities of OGM and ONT in Parkinson's disease.

**Methods:**

Ultra‐high molecular weight DNA was derived from blood and fibroblast cultures of 19 patients with mostly early‐onset Parkinson's disease, and used for Nanopore sequencing and optical genome mapping. The size distributions of deletions and insertions were compared, and variants were filtered for rare or potentially pathogenic variants in 134 known movement disorder genes.

**Results:**

Both methods identified SVs > 50 kb; however, optical mapping identified fewer structural variants (49,677) compared to Nanopore sequencing (94,400), but detected six times more in the range 50–80 kb. In general, it detected significantly larger deletions and insertions (*p* < 2.2 × 10^−16^). Both methods detected a benign intergenic deletion (195 kb) near *ITPR1*, and optical mapping validated a previously published 7‐Mb *PRKN* inversion. Small heterozygous deletions in *ATXN2*, *SUCLA2*, and *PNKD* detected by optical mapping were identified to be intronic by Nanopore sequencing. No causal variants were identified in movement disorder genes.

**Interpretation:**

Optical mapping can be a powerful first‐line method for detecting large structural variants, but it requires a high‐resolution method to refine breakpoint positions. Despite certain limitations, Nanopore sequencing was highly capable of detecting large variants independently and allows for a highly complementary assessment and validation of structural variation in combination with optical mapping.

## 1 Introduction

Structural variants (SVs) are one of the largest contributors to genetic variation between human genomes [1, 2] and are commonly defined as alterations that exceed 50 nucleotides in length. Subtypes include deletions, duplications, and insertions of genomic sequence, as well as balanced inversions and translocations, all of which can disrupt genes and regulatory elements, contributing to complex genomic rearrangements with potentially catastrophic consequences. Due to its high base‐calling accuracy, short‐read exome sequencing is widely applied in genetic research, particularly for detecting pathogenic single‐nucleotide variants (SNVs) or short indels [3, 4, 5]. However, its capabilities for detecting SVs of comparable size to the read length or even larger SVs are limited, especially affecting insertions or complex rearrangements [6, 7, 8, 9, 10]. In the field of neurodegenerative movement disorders, long‐read sequencing (LRS) methods like single‐molecule real‐time (SMRT) technology by Pacific Biosciences (PacBio) or Nanopore sequencing by Oxford Nanopore Technologies (ONT) have the potential to help fill in remaining gaps in our genetic understanding of these severe diseases [11, 12, 13]. In Parkinson's disease (PD) research, ONT was recently performed on *N* = 35 affected individuals with heterozygous variants in *PRKN* and *PINK1*, with the aim of identifying previously undetected secondary variants [14]. Among 23 heterozygous *PRKN* carriers, ONT identified six (~26.1%) with additional SVs in trans [14]. The functional consequence of a non‐coding African ancestry‐specific *GBA1* risk variant was identified using long‐read RNA sequencing despite the nearby *GBA1LP* pseudogene exhibiting high sequence homology (~96%) [11]. In the context of the first short‐read‐based SV GWAS in PD, further benchmarking using LRS revealed that ~30% of the identified SVs were false positives, and ~85% of SVs detected with long‐read sequencing could not be detected using short‐read sequencing [13, 15]. Finally, ONT was able to identify a large 7‐Mb inversion disrupting exon 12 of *PRKN* in compound heterozygous affected twins with early‐onset PD [16].

Optical genome mapping (OGM) is a different approach to whole‐genome sequencing that is more akin to classic cytogenetic tools, such as fluorescence in situ hybridization (FISH) and karyotyping, but greatly surpasses them in terms of resolution and sensitivity [17, 18]. It has been used to identify and size repeat expansions in *RFC1* that cause late‐onset ataxia [19, 20], demonstrating higher accuracy compared to Southern Blotting [19]. Despite its high accuracy, OGM is often applied to validate a detected variant rather than as a first‐line technique [21, 22]. One limitation of OGM is the lower resolution threshold of 500 bp compared to sequencing methods like ONT, which can capture single‐base pair changes. However, molecules imaged during OGM have a very high N50 of ≥ 150 kb and are measured to a coverage of ≥ 100 *X*, allowing for high sensitivity and confidence [23]. These large fragment sizes also allow for effective phasing, as recently demonstrated for large compound heterozygous *PRKN* deletions [24].

In this study, we performed the first genome‐wide comparison of the SV detection capabilities of ONT and OGM in non‐neoplastic human‐derived samples, focusing on patients with PD as a use case. We evaluated their concordance and complementarity genome‐wide and for potentially pathogenic variants in 134 known genes associated with movement disorders presented by the Movement Disorder Society (MDS) Taskforce [25] to benchmark their ability to identify different types and sizes of medically relevant SVs.

## 2 Methods

### 2.1 Research Cohort

**FIGURE 1:**
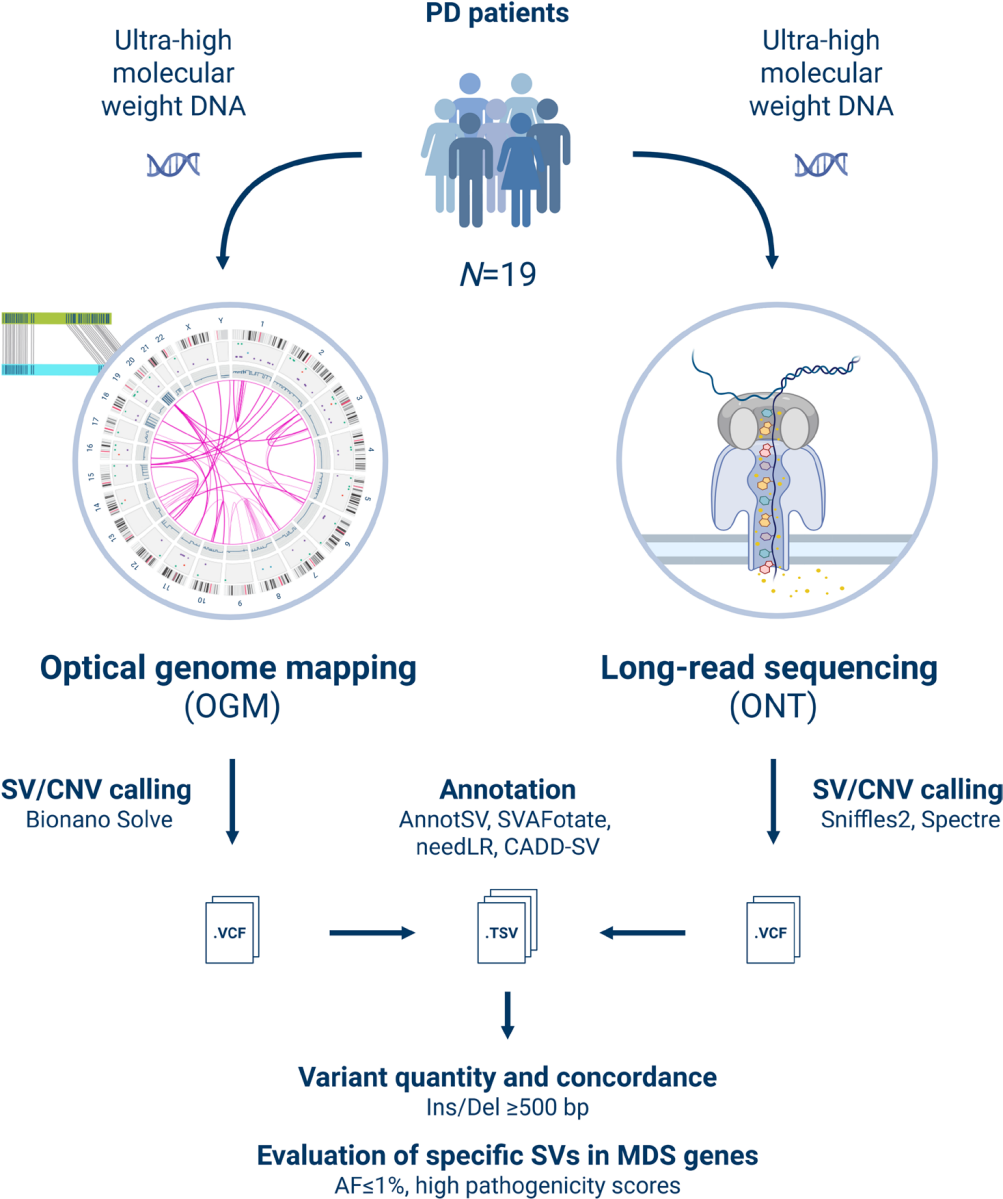
Overview of the analysis workflow. Whole blood and skin biopsies (to grow fibroblast cultures) were obtained from *N* = 19 predominantly early‐onset PD patients and used to isolate genomic ultra‐high molecular weight DNA (UHMW). The SV detection capabilities of ONT and OGM were compared. Created in BioRender. Fienemann, A. (2025) BioRender.com/uhc6n0i. AF, allele frequency; CNV, copy number variation; Del, deletion; Ins, insertion; MDS, Movement Disorder Society; SV, structural variant.

Nineteen patients affected by idiopathic PD were examined using OGM and ONT LRS (Figure 1). Fourteen were of central European descent, and five were of Tunisian origin and had a consanguineous family background (Table S1). In total, seven individuals had a positive family history of PD. Preferentially, patients exhibiting an early disease onset (average onset age ± standard deviation: 39 ± 14 years; range 14–73 years) were included to increase the likelihood of detecting a monogenic disease cause. All patients provided informed consent, and local ethics committee approval was obtained at the University of Lübeck and the University of British Columbia. As a separate case study outside of the cohort, OGM was performed on a single sample of a patient with PD originating from Japan, who carried a known 7 Mb inversion affecting *PRKN* [16].

### 2.2 Optical Genome Mapping

The OGM laboratory procedure is described in more detail in the supplement (Text S1). In brief, two blood samples and 17 skin biopsies were used to obtain genomic ultra‐high molecular weight DNA (UHMW), which was enzymatically labeled and imaged on the Bionano Saphyr. The Bionano Solve (v3.8) de novo pipeline processed the generated optical maps. Bionano Access software (v1.8) analyzed the results and exported the identified SVs and CNVs as VCF and SMAP files containing the variants.

### 2.3 Long‐Read Sequencing

The ONT laboratory procedure is described in more detail in the supplement (Text S2). In brief, LRS was performed at two different facilities: externally at the NGS Competence Center Tübingen (NCCT) (*N* = 7) and internally at the Institute of Neurogenetics (ING) Lübeck (*N* = 12). UHMW DNA was used for library preparation and sequencing on an ONT PromethION device. The base‐calling was performed using Dorado v0.6.3 (basecall server 7.4.12). The generated output files (.fastq) were used for in‐house analysis. PacBio LRS was performed for L‐3049, L‐8302, and 3049–10 to validate large outlying SVs (Text S3).

### 2.4 Data Analysis

The ONT fastq files containing the sequence information were concatenated and converted to an unmapped BAM file using v1.9 of Samtools [26]. This input was analyzed using v2.3.1 of the wf‐human‐variation workflow of EPI2ME Labs (github.com/epi2me‐labs/wf‐human‐variation), a collection of tools supported by ONT. This includes alignment to the GRCh38 reference genome using v2.28 of minimap2 [27] as well as variant calling using v2.6.2 of Sniffles2 for SVs [28, 29] and v0.2.1 of Spectre for CNVs [30]. The older v2.3.3 version of Sniffles2 was additionally applied to evaluate the improvement in large variant detection for specific variants. The Sniffles2 and Spectre variants, as well as the OGM variants detected by Bionano Solve, were annotated using v3.4 of AnnotSV [31] and v1.0 of SVAFotate [32] to obtain functional and regulatory information as well as population‐level allele frequencies based on v4.1.0 of the SV callset of the Genome Aggregation Database (gnomAD SV). To account for larger variants not present in the short‐read‐based gnomAD SV database, the variants were additionally annotated using v3.4 of needLR [33] to obtain allele frequencies based on the ONT LRS data from the 1KGP‐ONT Consortium (450 samples). V1.1.2 of CADD‐SV [34] was applied to score the potential effect of coding and especially noncoding SVs. The variants were assessed and visualized using R (v4.5.0) [35] with the packages data.table (v1.17.2) [36], tidyverse (v2.0.0) [37], dplyr (v1.1.4) [38], and ggplot2 (v3.5.2) [39]. Only variants with a Phred score of at least 20 (corresponding to 99% confidence and the maximum for OGM variants) or −1 (confidence cannot be assessed) were considered. Large SVs (> 50 kb) were examined to evaluate the performance of both methods in detecting large variants. All large deletions and insertions detected by ONT (Tables S10 and S11) and OGM (Tables S12 and S13) are included as Supporting Information. Wilcoxon signed‐rank tests were performed on the length distribution of deletions and insertions detected by both OGM and ONT to test for differences in the detected variant length. To estimate the concordance of both methods, ONT variants ≥ 500 bp (resolution of OGM) were compared to the OGM variants and matched according to the SV/CNV type, chromosome, position in the GRCh38 reference genome, and variant length. Insertion and duplication calls were combined into one category to account for different nomenclatures between variant callers [40]. As an approximation, a variant was considered a match if the difference in the SV's start, end and length did not exceed an interval of ±6 kb for variants ≤ 50 kb, ±15 kb for large variants, > 50 kb or ±7 kb for variants in movement disorder genes, based on the median uncertainty (95% CI) of the OGM variant calls (Table S2). The ONT variants served as the basis for the comparison, enabling better discrimination of individual close SVs due to the higher breakpoint accuracy of the Sniffles2 calls. Rare variants with a population allele frequency of ≤ 1% in either gnomAD SV or the internal Bionano database, variants with high pathogenicity prediction, for example, pathogenic according to the ACMG [41] or CADD‐SV ≥ 20, or variants in or near known movement disorder genes presented by the MDS Taskforce [25], were evaluated to compare the performance and concordance of both methods regarding the detection of novel specific variants (Figure S1). The total number of variants spanning exons was also further evaluated (Figure S2).

For the T2T‐CHM13v2.0 aligned case study sample, which carries a known 7‐Mb inversion affecting *PRKN* [16], the tool BCFtools/liftover [42] was used to perform a liftover to GRCh38.

## 3 Results

### 3.1 Molecule and Read Statistics

For each OGM run, on average 2,289,579 molecules with a mean length of 240.4 kb were imaged. A mean N50 of 237.5 kb and a mean coverage of 176.7 *X* were obtained. A mean label density of 15.9 labels per 100 kb was measured (Table 1), which is in the expected range of 14–17/100 kb for optical mapping [43]. With ONT, a mean of 7,229,440 reads with a mean length of 14.6 kb were sequenced with a mean coverage of 26.8 *X* (Table 1). A mean N50 of 25.8 kb was achieved with the 12 samples processed at the ING Lübeck, showing higher results (N50 > 25 kb) compared to the seven samples sequenced at the NCCT (N50 < 20 kb) (Table S5). A mean Phred quality score of 15.3 was obtained (Table 1).

**TABLE 1:** Molecule and read statistics. Overview of different metrics describing the molecules imaged during OGM and the reads sequenced during ONT sequencing of the cohort.

Bionano optical genome mapping						
	Total molecules	Total length (Mb)	Mean length (kb)	Molecule N50 (kb)	Label density (*x*/100 kb)	Coverage (*X*)
Mean	2,289,579	545,936	240.35	237.51	15.9	176.7
Median	2,160,768	512,296	239.67	235.61	15.9	165.9
SD	607,168	122,522	22.11	28.22	0.5	39.7

Oxford nanopore long‐read sequencing					
	Total reads	Mean *Q*‐score	Mean length (bp)	Read N50 (kb)	Coverage (*X*)
Mean	7,239,301	15.3	14,587	25.80	26.8
Median	6,065,873	14.9	16,036	31.84	24.6
SD	3,949,356	3.3	6936	11.44	6.3

*Note:* The cohort comprised *N* = 19 individuals.

Abbreviations: bp, base pairs; kb, kilobase; Mb, megabase; *Q*‐score, read quality (Phred); SD, standard deviation; *X*, depth of coverage.

### 3.2 Structural Variant Detection With OGM and ONT

Across all patients, OGM identified 77,119 SVs, corresponding to a mean of 4056 SVs per individual (Table 2). After filtering for high‐confidence variant calls (Confidence ≥ 99%, Phred quality score ≥ 20), a total of 49,677 variants remained. A total of 804 high‐quality variants showed a rare population allele frequency of ≤ 1% in the internal Bionano control database. There were 299 high‐quality variants in known movement disorder genes, four of which were rare.

**TABLE 2:** Quantity of SVs and CNVs. High‐quality SVs and CNVs identified with OGM and ONT, as well as filtered subsets for rare variants and variants in known genes associated with movement disorders.

Bionano optical genome mapping					
	All SVs, CNVs (unfiltered)	All SVs, CNVs (high QC)	SVs or CNVs in MDS genes	Rare SVs or CNVs	Rare SVs or CNVs in MDS genes
Total	77,119	49,677	299	804	4
Mean	4056	2597	15	40	*NA*

Oxford Nanopore long‐read sequencing					
	All SVs, CNVs	All SVs, CNVs (≥ 500 bp)	SVs or CNVs in MDS genes	Rare SVs or CNVs	Rare SVs or CNVs in MDS genes
Total	462,846	94,400	1844	30,531	96
Mean	24,360	4968	97	1607	5

*Note:* Variants were considered high‐quality with a Bionano confidence score greater than 0.99 (Phred quality score of 20). All ONT variants passed the quality criteria (FILTER = PASS) and exhibited a Phred quality score of ≥ 20. Variants with an allele frequency of ≤ 0.01 in the internal Bionano control database or the gnomAD 4.1 SV database were considered rare. Rare: AF ≤ 0.01 in the internal Bionano control database or gnomAD SV 4.1.

In the ONT data, the SV caller Sniffles2 and the CNV caller Spectre identified 462,846 SVs and CNVs across all samples with a mean number of 24,360 per individual. All variant calls by Sniffles2 met the quality cutoff and showed a Phred quality score of at least 20. Across all variants detected with ONT, 94,400 were larger than 500 bp (resolution threshold of OGM). Subsequently, 1844 variants were in known genes associated with movement disorders. Considering population variant frequency, 30,531 variants showed at least 50% overlap with a gnomAD SV database entry (Overlap fraction product ≥ 50%) and a rare population allele frequency of ≤ 1%. Finally, a mean of five variants per sample was both rare in the gnomAD SV database and was found in genes implicated in movement disorders (96 in total over all samples).

### 3.3 Fractions of Structural Variant Types and Length Distribution

**FIGURE 2:**
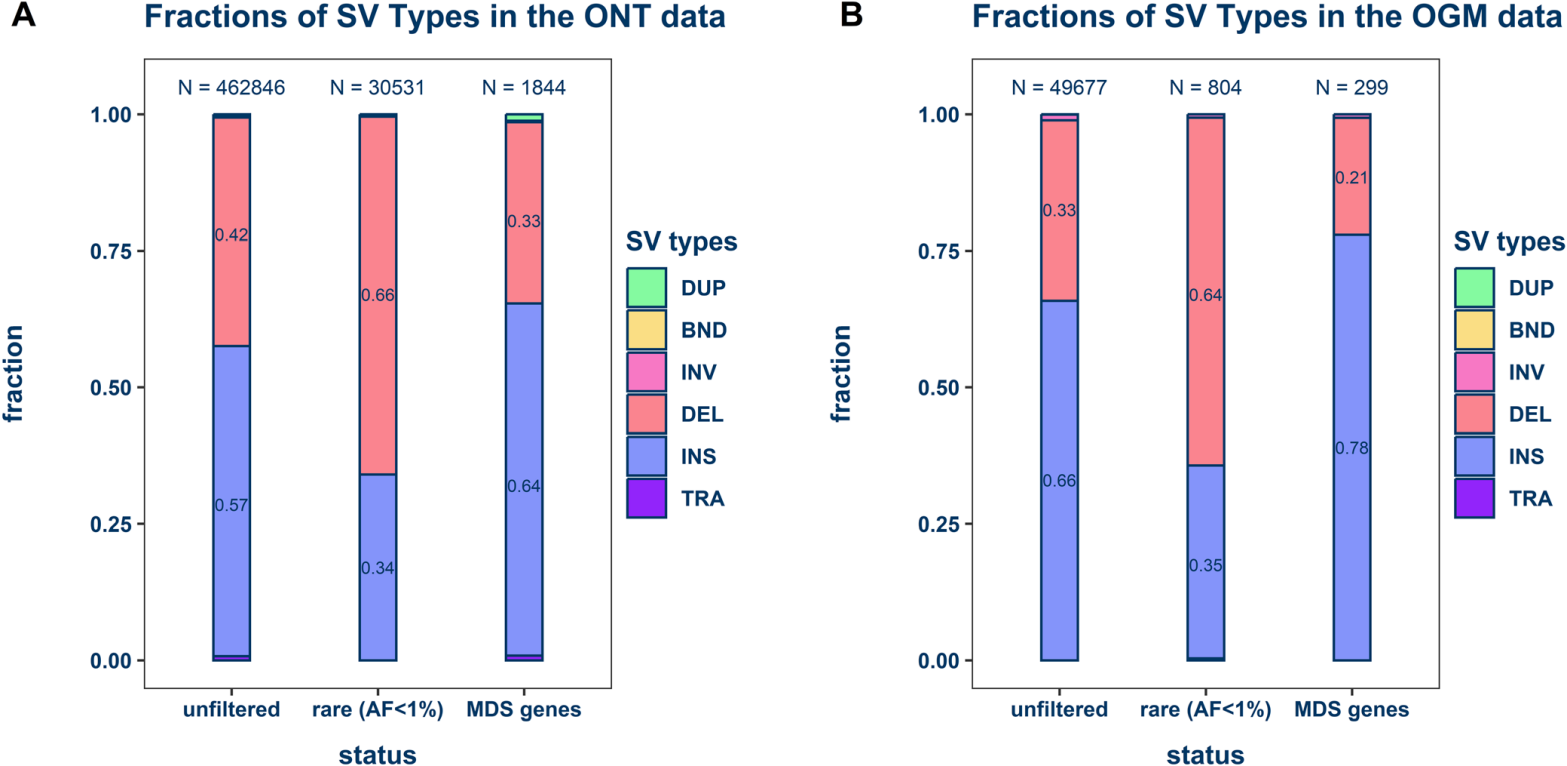
Fraction of SV and CNV types. The stacked bar plots show the fraction of duplications (DUP), break‐ends (BND), inversions (INV), deletions (DEL), insertions (INS), and translocations (TRA) in all high QC SVs and CNVs identified by OGM and ONT LRS and in a subset of variants overlapping with known genes associated with movement disorders. (A) Unfiltered (but high QC) and MDS variants identified in the ONT data. (B) Unfiltered (but high QC) and MDS variants identified in the OGM data.

The fractions of SV types detected by OGM and ONT vary among the different filtered subsets (Figure 2). For both methods, the detected SVs are predominantly deletions and insertions, with a combined fraction of 97.7%–99.5% in all subsets. Duplications (0.0%–1.2%), inversions (0.0%–1.0%), and translocations (0.0%–0.9%) are less represented (Table S3).

The high‐confidence variants detected by OGM consist of 32,706 (65.8%) insertions and 16,422 (33.1%) deletions (Figure 2B). In comparison, 284 (35.3%) insertions and 512 (63.7%) deletions are in the rare subset. The variants in movement disorder genes [25] consisted of 233 (78.0%) insertions and 64 (21.4%) deletions. ONT detected 262,935 (65.8%) insertions and 193,453 (41.8%) deletions (Figure 2A). In comparison, the subset of rare variants consists of 10,392 (34.0%) insertions and 20,005 (65.5%) deletions. The variants in genes implicated in movement disorders comprise 1189 (64.5%) insertions and 612 (33.2%) deletions. The fraction of deletions in the rare subset was overrepresented for both OGM (from 33.1% to 63.7%) and ONT (from 41.1% to 65.5%).

**FIGURE 3:**
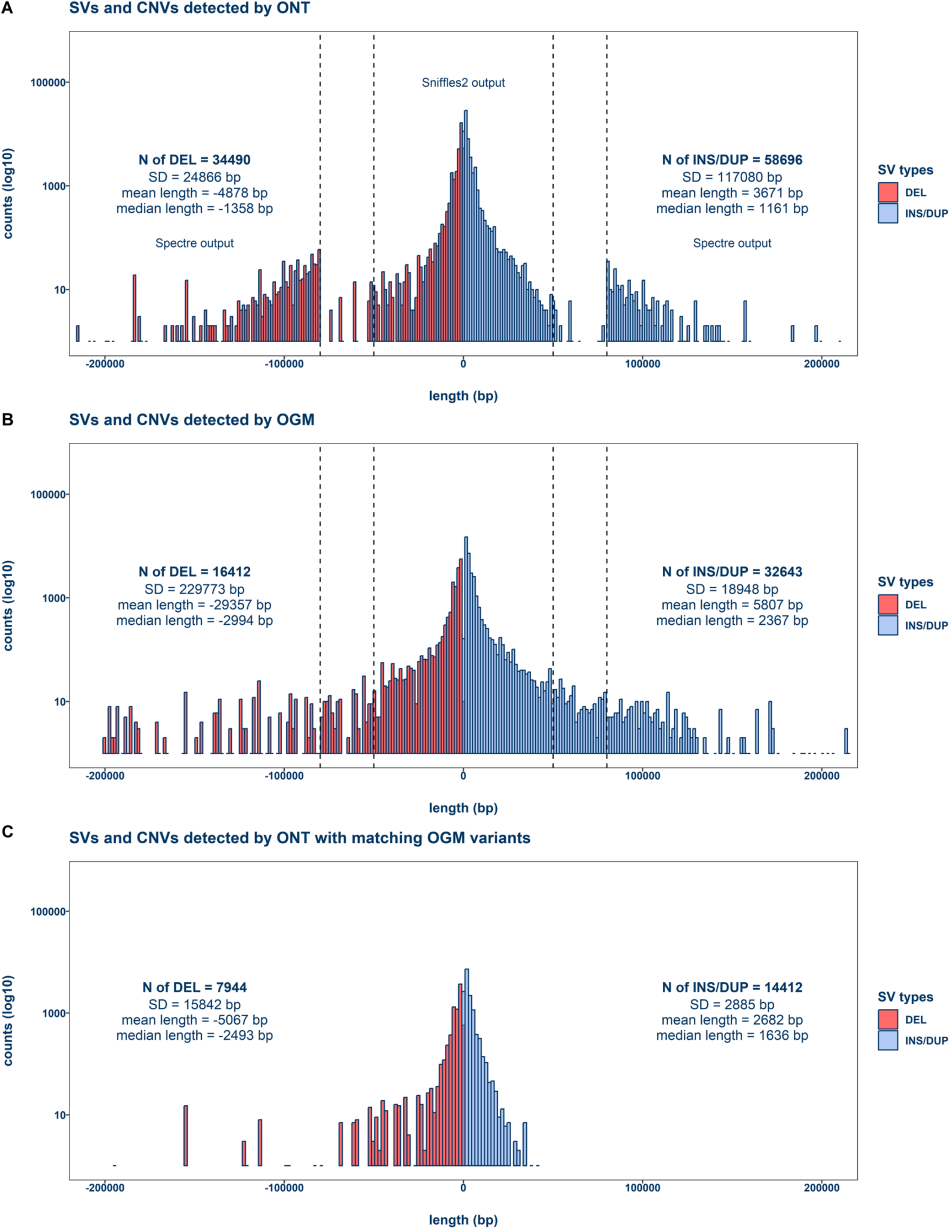
Length distribution of SVs and CNVs. The mirrored histograms show the length distribution of deletions and insertions/duplications ≥ 500 bp called by ONT and OGM. The variant length in base pairs (bp) is plotted against the logarithmic counts of the variants. (A) SVs and CNVs identified by Sniffles2 and Specter in the ONT data. (B) SVs and CNVs identified by Bionano Solve in the OGM data. (C) Variants called by ONT with overlapping calls by OGM. DEL, deletion; DUP, duplication; INS, insertion.

Both methods display a distinct length distribution of all insertions and deletions exceeding 500 bp (Figure 3). In the distribution of the ONT variants (Figure 3A), the variants called by Sniffles2 are, except for a small number of very large SVs, mostly confined to smaller sizes (≤ 50 kb). Spectre called larger CNVs starting at approximately 80 kb. Only 58 deletions and nine insertions are detected in the length bracket of approximately 50–80 kb. This visible detection gap is not present in the distribution of the OGM variants. OGM detected 384 SVs in the range 50–80 kb, including 37 deletions detected by ONT (Figure 3B). OGM detected a mean deletion length of 29,357 bp, approximately six times larger than that of the ONT variants (4878 bp). The standard deviation was higher for the OGM variants (±229,773 bp compared to ±24,866 bp), and the median deletion size of the OGM calls was about twice that of the ONT variants (2994 bp compared to 1358 bp). The mean insertion length of the ONT variants was slightly shorter (3671 bp compared to 5807 bp), and the median was approximately half the length (1161 bp compared to 2367 bp). The standard deviation (SD) of the ONT insertion lengths was higher (±117,080 bp compared to ±18,948 bp). Across all variant types, OGM detected fewer SVs (49,677) with a slightly larger mean size of 25 kb (median = 2.6 kb; SD = 209 kb) compared to ONT (94,400; mean = 7 kb; median = 1.2 kb; SD = 195 kb).

**FIGURE 4:**
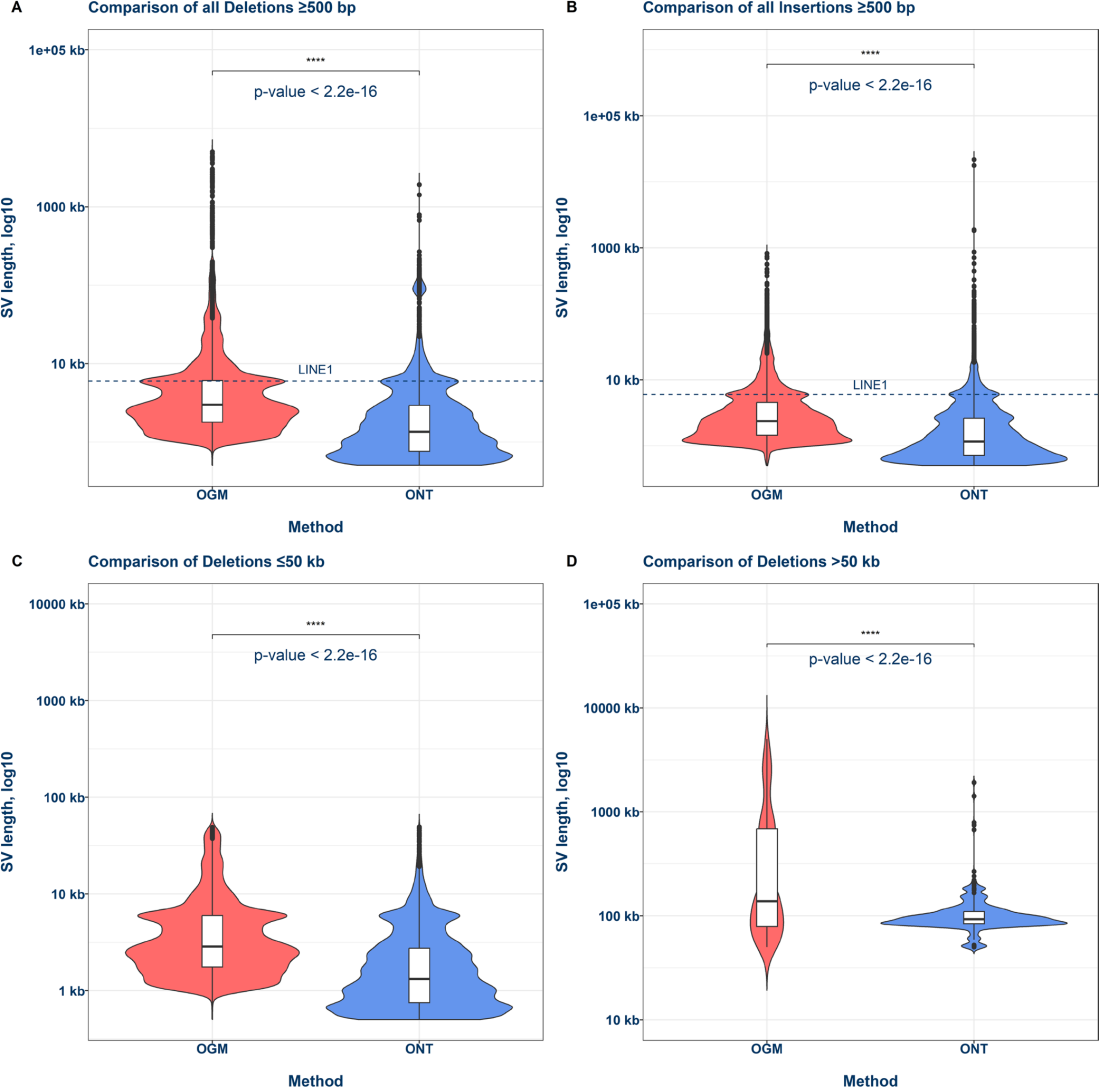
Comparison of SV and CNV lengths. Violin plots and box plots (median) illustrate the length distribution of high‐QC (Phred score ≥ 20) SVs and CNVs detected by OGM and ONT. A Wilcoxon signed‐rank test was performed using the SV length vectors of both methods (^****^ *p*‐value ≤ 0.0001). (A) Comparison of all deletions over the size of 500 bp. (B) Comparison of all insertions over the size of 500 bp; no insertions over 50 kb were detected by ONT. (C) Comparison of all deletions ≤ 50 kb. (D) Comparison of all deletions > 50 kb. The dashed line indicates a peak of LINE1 mobile elements near ±6 kb.

Wilcoxon rank‐sum tests on the length distributions of both methods (Figure 4) revealed that insertions and deletions detected by OGM were significantly larger than those detected by ONT (*p* < 2.2 × 10^−16^). An empirical cumulative distribution function (ECDF) of the distributions further illustrates this, as the OGM function is shifted towards longer SV lengths for all insertions and deletions (Figure S3). A characteristic peak of LINE1 mobile elements at approximately 6 kb [44, 45] is visible for both deletions and insertions.

The number of large (> 50 kb) ONT deletions and insertions with an overlapping fraction of 50% and an existing gnomAD SV frequency was 285/1037 (27.5%), while 55/1037 (5.3%) showed a matching entry in the 1KGP‐ONT samples. Only 15/1146 (1.3%) of all large (> 50 kb) OGM variants were not previously detected and thus not present in the internal Bionano database. Twelve were identified in the Nanopore data using the Integrative Genomics Viewer (IGV), including two deletions in 3049‐10, which PacBio LRS validated. Three were located in regions of segmental duplications or other hard‐to‐map regions and could not be identified in IGV (Table S14).

Most deletions greater than 50 kb detected by ONT are CNVs of a length of approximately 80–250 kb, with a small number of six very long (ranging from 0.6 to 1.9 Mb) deletions (Figure 4A,D). One 750 kb deletion was also detected using OGM (95% CI of ±10,088 bp). PacBio LRS did not validate any of the two deletions detected in L‐8302 and 3049‐10. Manual inspection of the remaining deletions using IGV revealed them to be artifacts, with three of them being located in areas masked for CNV calling in Bionano Solve, and none of them being found in the 1KGP‐ONT Samples (Table S8). Spectre and Sniffles2 identified 253 and 50 duplications over 50 kb, respectively, in the size range of up to 1 Mb, similar to the OGM insertions (Figure 4B). Three duplications were large outliers, ranging from 1.8 to 21 Mb (Figure 4B), that were also considered artifacts after IGV inspection (Table S8). Sniffles2 called 12 clear insertions > 50 kb in the ONT data, including the aforementioned nine insertions in the length bracket of 50–80 kb. All of them were only supported by a single read, and PacBio LRS did not validate any of the insertions detected in L‐8302 and 3049‐10 (Table S9).

### 3.4 Concordance Between OGM and ONT

**FIGURE 5:**
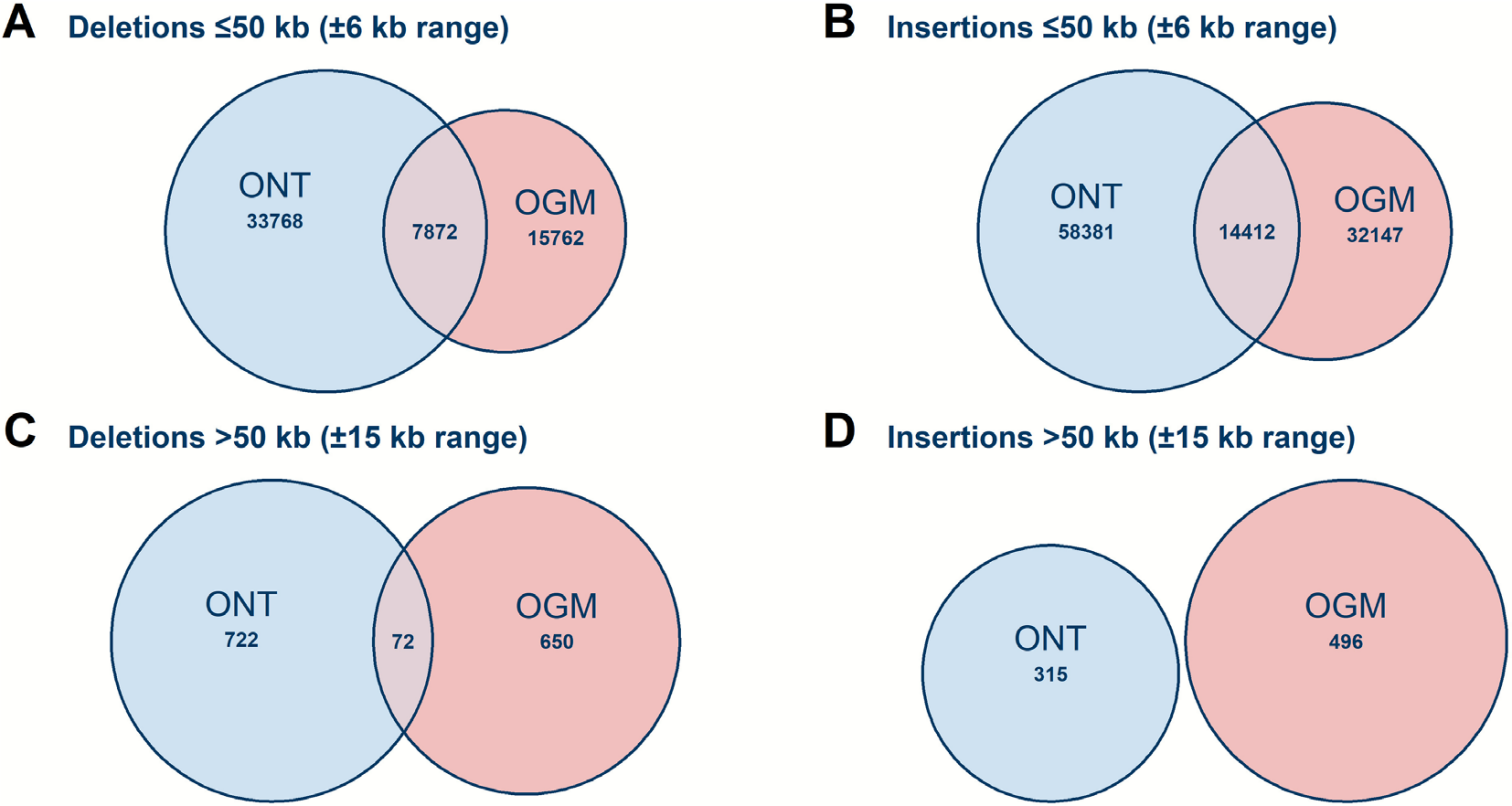
Concordance of variants between ONT and OGM. The Venn diagrams show the approximate overlap between small (> 500 bp and ≤ 50 kb) and large (> 50 kb) deletions (A, C) and insertions (B, D) identified by ONT and OGM. Due to the difference in methodology, the SVs in the overlap fraction are not exact matches. A fixed interval (±6 and 15 kb) regarding the start and end of the variants was applied based on the median 95% confidence interval of the OGM calls (Table S2).

Using a fixed interval of 6 kb for small (≤ 50 kb) and 15 kb for intermediate/large (> 50 kb) deletions and insertions, the concordance of OGM and ONT is low (Figure 5). About twice as many small deletions (ONT: 33,768; OGM: 15,762) and insertions (ONT: 58,381; OGM: 32,147) were called by ONT compared to OGM, with 7872 deletions (23.3%) and 14,412 insertions (24.7%) having a matching entry in the OGM variants (Figure 5A,B). In the context of large variants (> 50 kb), a similar number of deletions was called by OGM and ONT (ONT: 722; OGM: 650), while OGM called slightly more insertions (ONT: 315; OGM: 496). Only 72 large deletions (10%) detected by ONT had a matching entry in the OGM data, while no large insertions showed overlap between both methods (Figure 5C,D). In total, 147 (46.6%) out of the 315 large insertions (including 303 duplications) and 278 (38.5%) out of the 722 deletions detected by ONT could not be confirmed via visual inspection in IGV.

Out of the ONT variants, 5107 deletions (14.8%) and 14,151 insertions (24.11%) were identified as rare (occurring only once or less) by needLR in the 1KGP‐ONT database. Regarding the variants detected by both methods, 441 deletions (5.6%) and 2894 insertions (20.1%) were considered rare.

The variants detected with both methods (Figure 3C) are predominantly small, with a mean length of 5067 bp for the deletions and 2682 bp for the insertions (SD = 15,824 bp and 2885 bp, respectively). However, except for a large 750 kb deletion, the remaining 71 large deletions found by both methods (Figure 5C) are visible as discrete bins in the histogram. Both methods detected a similar number of deletions (ONT: 82, OGM: 64) and insertions (ONT: 285, OGM: 232) in known genes associated with movement disorders (Figure S4). Out of the ONT calls, 44 deletions (53.7%) and 59 insertions (20.7%) have a matching entry in the OGM variants.

### 3.5 ONT Refines Variant Positions in Movement Disorder Genes

**FIGURE 6:**
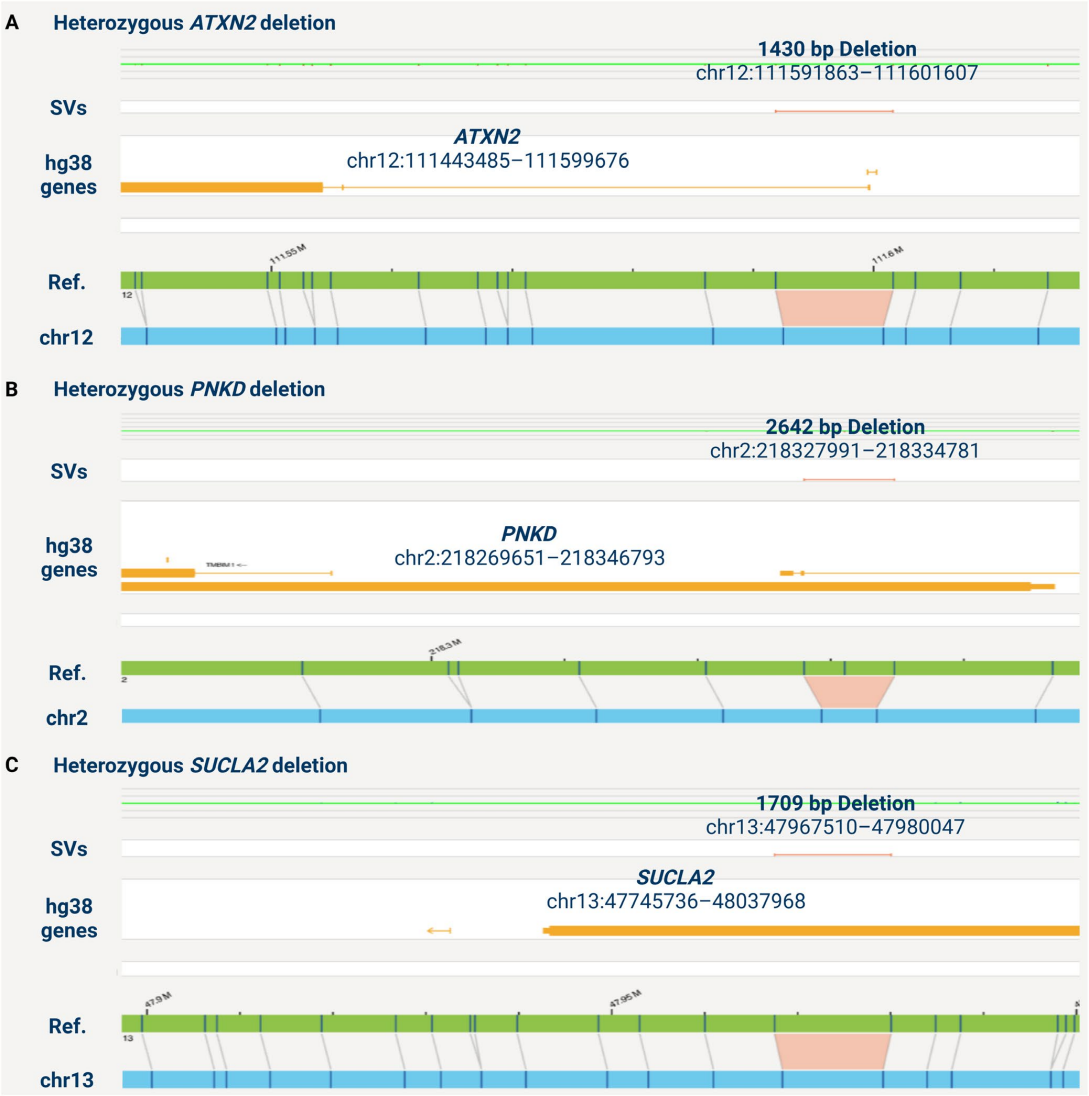
Bionano Access snapshots of three deletions affecting MDS Taskforce genes. The figure shows three small heterozygous deletions in known genes associated with movement disorders identified by both OGM and ONT. All of them lie in intronic regions. (A) A 1.4‐kb deletion in the individual L‐3047 affecting the *ATXN2* gene. (B) A 2.6‐kb deletion in 3048‐28 affecting *PNKD*. (C) A 1.7‐kb deletion in L‐3049 affecting *SUCLA2*. Annotated in BioRender. Fienemann, A. (2025) BioRender.com/jclzn7w.

Specific variants were evaluated in more detail to highlight and compare the aptitudes of both methods. Three small heterozygous variants in the movement disorder genes *ATXN2*, *SUCLA2*, and *PNKD* showed a rare allele frequency in the internal Bionano control database (Figure 6). OGM detected the heterozygous 1.4 kb deletion in *ATXN2* (Figure 6A) at the position chr12: 111,591,863–111,601,607 with an uncertainty (95% CI) of ±4157 bp. This region spans exon 1 of the *ATXN2* gene. OGM detected the 2.6 kb deletion in *PNKD* (Figure 6B) at the position chr2: 218,327,991–218,334,781 with an uncertainty (95% CI) of ±2074 bp. This region only spans intronic sequence. The heterozygous 1.7 kb deletion in *SUCLA2* (Figure 6C) was detected by OGM at the position chr13: 47,967,510–47,980,047 with a 95% confidence interval (CI) of ±5414 bp. This region overlaps with exons 5 and 6 of *SUCLA2*. All three variants were also identified by ONT (Figures S5–S7) with high precision (95% CI of ±0 bp) and were found to be rare in the 450 current samples of the 1KGP‐ONT consortium; only the *ATXN2* SV was found in one sample of the database (HG01372). Yet, ONT specified all three variants as affecting only intronic sequences. In general, OGM identified 14,036 deletions and insertions (28.3% of all structural variants) in exons, of which ONT detected 3267. Among these, ONT specified only 372 variants (11.4%) to span coding regions (Figure S2).

**FIGURE 7:**
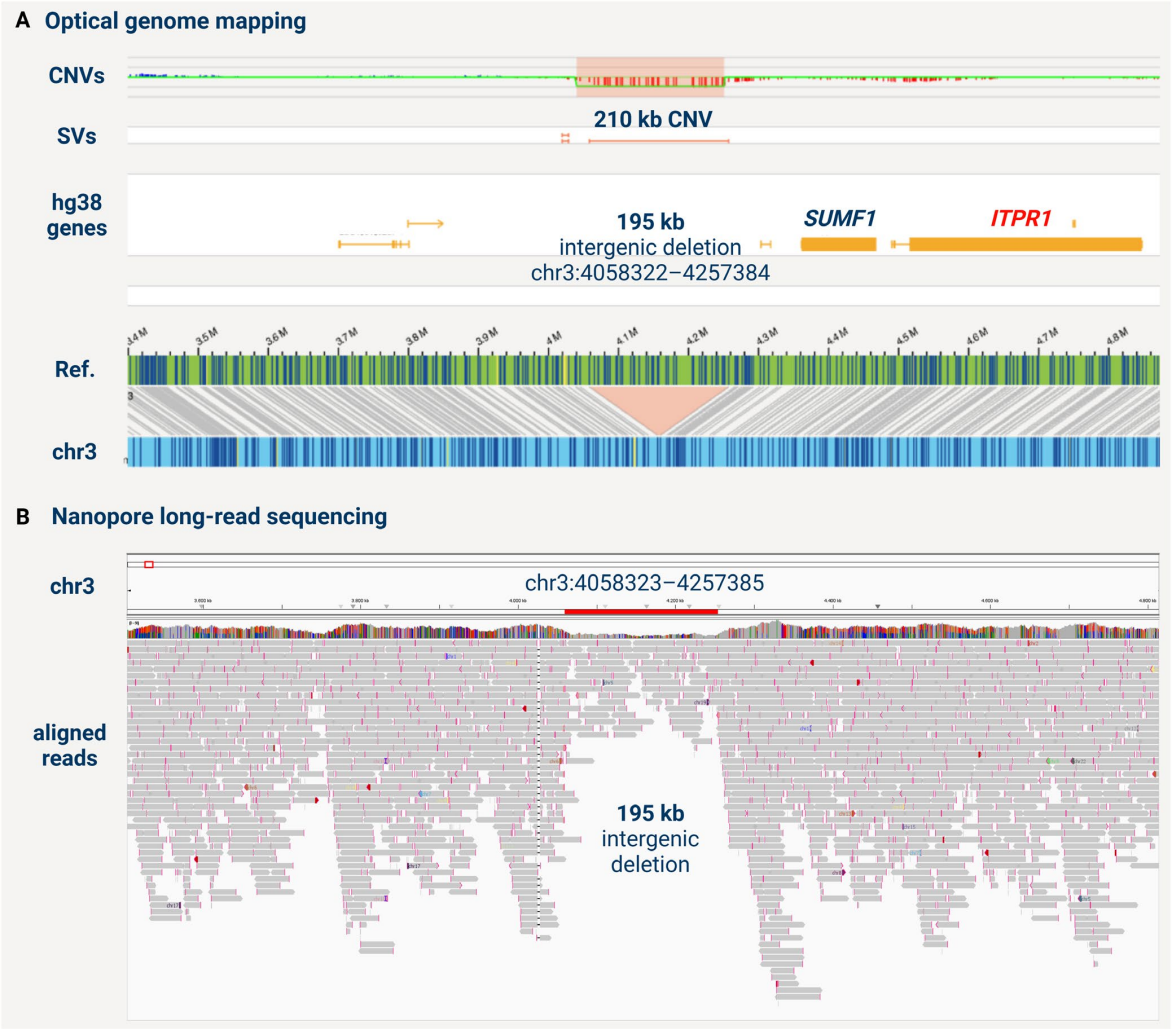
A 195‐kb intergenic deletion. The figure shows a heterozygous 195‐kb intergenic deletion near *ITPR1*, detected by both OGM and ONT. (A) A Bionano Access snapshot of the variant, showing a reduced copy number in the same region. (B) A snapshot from the Integrative Genomics Viewer (IGV) displaying ONT reads mapped to the GRCh38 reference genome. Annotated in BioRender. Fienemann, A. (2025) BioRender.com/xakay3y.

Both OGM (Figure 7A) and ONT (Figure 7B) identified a large heterozygous deletion of 195 kb in an intergenic region near the gene *ITPR1*. OGM detects the variant with an uncertainty (95% CI) of ±1972 bp, and an apparent reduction in the copy number is observed in the same area. The variant showed an allele frequency of < 1% in the internal Bionano SV database, gnomAD SVs v4.1.0, and the 450 current samples of the 1KGP‐ONT consortium. A PHRED‐scaled CADD‐SV score of 21.1 was obtained for the variant, indicating that it is in the top 1% of variants in the gnomAD‐SV score distribution.

### 3.6 Case Study: Large PRKN Inversion Detection by OGM

**FIGURE 8:**
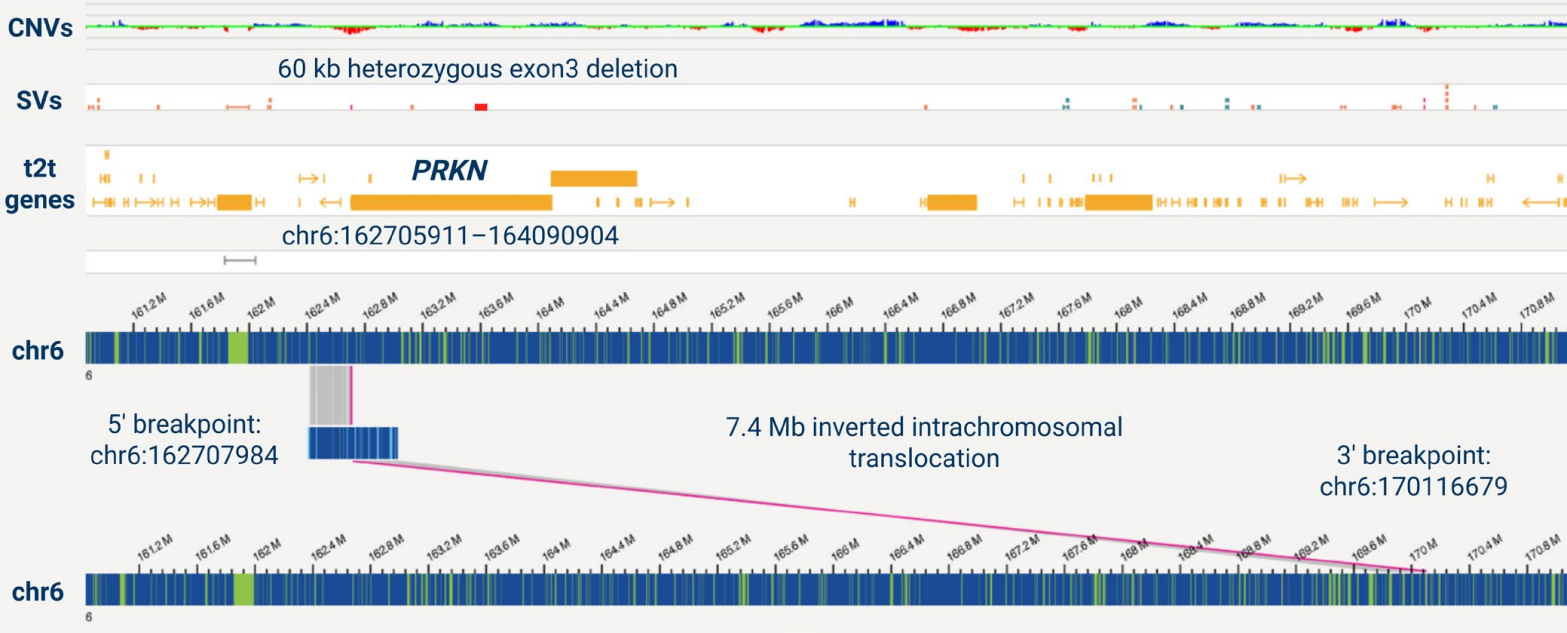
Bionano Access snapshot of a 7‐Mb inversion affecting *PRKN*. The figure shows a 7‐Mb inversion originally discovered in monozygotic twins with young onset dystonia‐parkinsonism [16]. Both the large inversion and the exon 3 *PRKN* deletion in trans are detected by optical genome mapping. Due to the size of the variant, it is called an inverted intrachromosomal translocation. Annotated in BioRender. Fienemann, A. (2025) https://BioRender.com/njab828.

Finally, a sample with a previously reported 7‐Mb inversion affecting *PRKN*, detected using ONT in two monozygotic twins with young‐onset dystonia‐parkinsonism [16], was analyzed using OGM to test the method's capabilities further. An inverted intrachromosomal translocation affecting exon 12 of *PRKN* and an exon 3 deletion in trans were detected (Figure 8). The breakpoints of the translocation were detected at the GRCh38 positions 5′: chr6: 161,349,490 (95% CI of ±7670 bp) and 3′: chr6: 168,770,610 (95% CI of ±6448 bp). The *PRKN* deletion was detected at the position chr6: 162,205,394–162,278,131 (95% CI of ±6306 bp).

## 4 Discussion

In this study, we compared the SV detection capabilities of ONT and OGM in the whole genomes of *N* = 19 idiopathic PD patients, evaluating genome‐wide concordance and complementarity, as well as their ability to detect potentially pathogenic variants in known movement disorder‐associated genes.

Both methods identified a total number of SVs per genome within the previously reported range of ~20,000 SVs and CNVs for ONT [33, 46] and ~5000 for OGM [17, 47], which primarily consisted of insertions and deletions. Insertions accounted for the largest percentage of all variants detected by OGM and ONT (approximately 60%). However, their fraction dropped by about half in the filtered subset of rare variants, like for the ONT variants with a gnomAD SV allele frequency ≤ 1%. As the gnomAD database is based on short‐read sequencing, this could indicate its limited capacity to detect insertions compared to deletions. A recently published effort to sequence 1019 population‐diverse human genomes using ONT revealed that 50.9% of identified insertions were absent in gnomAD SV [48]. In contrast, only 14.5% of deletions were not previously represented in the short‐read‐based database. Sequencing‐based approaches generally struggle with large insertions, as they disrupt accurate read alignment [10] and pose unique challenges for SV calling algorithms, for example, due to junctional homologies at the breakpoints [49]. LRS has improved SV detection accuracy compared to short‐read sequencing, particularly in the detection of insertions [6, 13], highlighting the utility of technology‐matched SV catalogs, such as the 1KGP‐ONT Consortium. While these hurdles also apply to OGM, the high coverage (> 150 *X*) and long mean molecule length (> 200 kb) of the measured molecules even enabled the detection of very large insertions, as demonstrated by the comparison of SV and CNV lengths between the two methods.

For all deletions and insertions, the OGM‐detected variants were significantly longer than those detected by ONT (*p* < 2.2 × 10^−16^). On the other hand, ONT detected a considerably larger number of variants. In addition to small SVs and Indels under the resolution limit of OGM, twice the number of deletions and insertions exceeding 500 bp were detected compared to the stringently filtered OGM variants. Still, false positives exist and 38.5%–46.6% of large (> 50) deletions and insertions were not confirmed in IGV. While a fraction of the smaller variants could be attributed to artifacts as well, due to limited base‐calling accuracy, ONT is highly capable of detecting small and intermediate‐length SVs with high precision [12, 14, 50].

The length distribution of the ONT variants illustrates the benefit of performing read depth‐based CNV calling, in addition to SV calling by Sniffles2, which was previously known to be limited in detecting larger variants (> 50 kb) [51]. However, there are exceptions, such as the heterozygous 195‐kb intergenic deletion near the ataxia gene *ITPR1* [52, 53], which OGM detected and ONT validated with single‐base pair accuracy (95% CI of ±0 bp). The phred‐scaled CADD‐SV score of > 20 indicates a moderately predicted deleteriousness of the variant, potentially due to the spanning of cis‐regulatory elements. However, the patient did not show clinical signs of spinocerebellar ataxia type 15 (SCA15).

A detection gap remains in the length bracket of approximately 50–80 kb for both insertions and deletions detected by ONT, as CNV calling by Spectre is optimized for variants > 100 kb. OGM detected six times more variants in this range and is currently complementary for covering this gap.

The widespread adoption of LRS has led to steady improvements in variant callers, with the new 2.6.2 version of Sniffles2 now being able to detect a heterozygous 98‐kb deletion spanning the *PEX7* gene previously only detected by OGM (Figure S8). Using v2.3.3 of Sniffles2 or using Specter, ONT did not detect the SV (Figure S9) as it falls outside the optimal detection range of both. However, using v2.6.2 of Sniffles2, the variant was detected with high precision (95% CI of ±0 bp) (Figure S10).

Previous studies compared SVs detected by ONT and OGM in SKBR3 human breast cancer cells [54], the plant genome of Arabidopsis thaliana [55], 234 individually curated variants in *N* = 9 parent–child trios [6], and renal cell carcinoma tumor tissue [56]. All of these studies reported a generally high degree of overlap between the two methods, as well as some discrepancies in SV detection due to differences in methodology and aptitude for different variant types and lengths, suggesting a synergy between the two approaches. However, to our knowledge, no previous study has compared the SV detection of ONT and OGM genome‐wide in non‐neoplastic human‐derived samples. In our study, the concordance between OGM and ONT was low, especially for large variants (> 50 kb), with 10%–24.7% of ONT variants matching those in the OGM dataset. There was also a decrease in the percentage of rare 1KGP‐ONT deletions (from 15.8% to 5.6%) in the concordant subset compared to the unique subset, which was not observed for the insertions (24.1% and 20.1% respectively). This could indicate that slightly more common deletions were detected by both methods. In the most recent comparison of both methods in renal cell carcinoma, the highest overlap was reported for the 1–5‐kb length bracket, with 1487 (81.7%) SVs being concordant [56]. ONT and OGM detected 1821 and 1679 SVs, respectively. For SVs ≥ 10 kb, OGM detected 232 SVs, compared to 48 detected by ONT, with 40 (~83.3%) being concordant [56]. However, only SVs called by Sniffles2 and no CNVs identified by Spectre or other methods were compared. In our study, CNVs were included, and 822 of 1037 deletions and insertions exceeding 50 kb were CNVs called by Spectre. This resulted in lower concordance between OGM and ONT due to the reduced breakpoint accuracy (1000 bp resolution) and a higher false‐positive rate of the read‐depth approach. Different versions of base‐calling models and variant detection tools for the ONT data also contribute to differences between our study and previous comparisons, for example, Guppy base‐calling instead of Dorado [6]. Still, a limitation of the here performed comparison is that while a fixed interval regarding the breakpoint positions provides a high‐level overview, not all nuances are captured, for example, subtle differences of smaller SVs or complex loci.

Catalogs of challenging and medically relevant genes, such as the ACMG [57] or other reported gene lists, for example, the Genome in a Bottle challenging medically relevant genes benchmark set of 217 SVs [58, 59], were helpful in the past to highlight the power of long‐reads to overcome repetitive gene regions and pseudogene homology [60]. Here, we studied the ability of OGM and ONT to identify medically relevant variants in known genes associated with movement disorders presented by the MDS Taskforce [25]. Both methods detected the same three rare and heterozygous deletions in the genes *ATXN2*, *SUCLA2*, and *PNKD*. *ATXN2* is implicated in spinocerebellar ataxia [61] but can also present with predominant Parkinsonism [62], while *PNKD* can cause paroxysmal non‐kinesigenic dyskinesia [63]. Both show autosomal dominate inheritance. *SUCLA2* can cause complex dystonia [64, 65] with autosomal recessive inheritance. Although OGM detected all three variants with high confidence, the uncertainty in the exact breakpoint positions prevented practical interpretation of the variants, as it was unclear if the coding sequence was involved or not. ONT finally identified all three to be intronic, emphasizing the advantage of single‐basepair resolution for interpreting, especially smaller SVs.

In the Japanese *PD* sample, OGM detected both the previously identified 7.4‐Mb inverted intrachromosomal translocation and 60 kb deletion in trans [16] affecting the early‐onset PD gene *PRKN* [66]. This aligns with previous observations that the Bionano Solve software represents inversions exceeding 5 Mb due to their size as intrachromosomal translocations [67]. OGM was able to phase the known inversion and deletion in trans and validate the compound heterozygous disruption of *PRKN* in a straightforward manner, confirming that OGM excels in detecting large and complex SVs. However, while OGM detected the 5′ breakpoint in exon 12, in line with the ONT results, the uncertainty of the position (±7.7 kb) leaves open the possibility that the breakpoint lies outside of the gene. Both cases reveal a limitation of OGM, being the breakpoint accuracy, which showed high variance depending on the local label density, with a median 95% CI of 15 kb (SD = 41 kb) for variants greater than 50 kb. As only 11.4% of coding OGM variants with a match in ONT were specified to span coding regions, applying a (targeted) sequencing method is advised to fine‐map the breakpoints of potentially pathogenic variants.

While this makes OGM less attractive in the context of efficient genetic testing, in this study, running a single sample with ONT was twice as expensive as OGM, with similar expertise and computational resources required for data analysis. ONT has the advantage that SNVs and small indels below the OGM resolution limit of 500 bp can be assessed in parallel with SVs. This is highlighted by the fact that only 6/96 (6.3%) of rare ONT variants in MDS genes were larger than 500 bp (Table S7). However, current variant callers also produced many false‐positive calls for large variants, complicating downstream analysis and necessitating visual inspection and validation of SVs, for example, by PCR.

The study of SVs has great potential to unravel the missing genetic factors and monogenic causes in the field of PD and other neurodegenerative disorders. Technologies like LRS and OGM provide us with the means to fill the gaps left by established methods, such as NGS [68, 69]. This is highlighted in a recent study where six individuals with unsolved SVs were analyzed using both ONT and OGM to resolve breakpoint junctions at the single‐basepair level and allow for effective variant phasing to unravel complex rearrangements [22]. There are limitations to address. While we were able to evaluate the validity of a number of large variants using PacBio LRS, there is a lack of widespread validation by an orthogonal method. Both different sequencing protocols (R9 and R10) and biomaterials (blood and fibroblasts) introduced variance in the variants detected by ONT.

Nevertheless, our study shows that OGM has a strong aptitude for detecting large and complex variants, while ONT excels at characterizing smaller to intermediate‐sized variants. Their combined application offers a complementary approach to enable broad detection and robust validation of structural variation.

## Author Contributions

A.F. contributed to the conceptualization of the study, data curation, formal analysis, methodology, investigation, software, visualization, writing of the original draft, and final approval of the manuscript. T.L. was involved in conceptualization, data curation, methodology, software, supervision, and final approval of the manuscript. S.S. participated in the methodology, investigation, and final approval of the manuscript. C.G. and M.M. contributed to methodology, data curation, software, and final approval of the manuscript. H.B. and K.L. contributed to funding acquisition and final approval of the manuscript. J.A.G. and D.E.M. contributed to the provision of software resources and final approval of the manuscript. K.D., M.F., N.H., S.B.S., F.H., and M.J.F. provided patient samples, clinical information of patients, and read and approved the final manuscript. K.K.U. participated in the investigation, software, and final approval of the manuscript. C.K. contributed to the conceptualization, project administration, funding acquisition, provision of resources, and final approval of the manuscript. J.T. was involved in conceptualization, funding acquisition, project administration, resource provision, supervision, and final approval of the manuscript.

## Funding

This research was funded by the Deutsche Forschungsgemeinschaft (DFG, German Research Foundation) (TR 1714/4‐1 and TR 1714/7‐1) and a Heisenberg Grant (TR 1714/8‐1) to J.T. H.B. and M.M. acknowledge funding by the DFG under Germany's Excellence Strategy—“EXC 22167‐390884018.”

## Ethics Statement

Written informed consent was obtained from all individuals and approved by the Ethics Committee at the University of Lübeck, Lübeck, Germany, and the University of British Columbia, Vancouver, Canada.

## Conflicts of Interest

J.T. has received travel funding to speak on behalf of ONT. C.K. has served as a medical advisor to Centogene, Takeda, and Biogen and received speakers' honoraria from Bial, as well as royalties from Oxford University Press and Springer Nature. D.E.M. is on scientific advisory boards at ONT and Basis Genetics, is engaged in research agreements with ONT and PacBio, has received research and travel support from ONT and PacBio, holds stock options in MyOme and Basis Genetics, and is a consultant for MyOme. J.A.G. has received travel support from ONT. The other authors declare no competing interests.

## Supporting information


**Data S1:** acn370332‐sup‐0001‐DataS1.docx.

**Table S1:** acn370332‐sup‐0002‐Tables.xlsx.

## Data Availability

The datasets used and/or analyzed during the current study are available from the corresponding author on reasonable request.
