# Supplementary material for: Complementarity of Long‐Reads and Optical Mapping in Parkinson's Disease for Structural Variants
Source: Ann Clin Transl Neurol. 2026 Feb 7;13(7):1467–81. doi: 10.1002/acn3.70332 (PMC13358575; doi:10.1002/acn3.70332)
Supplement: Supplementary file 1 — Data S1: acn370332‐sup‐0001‐DataS1.docx. [file ACN3-13-1467-s002.docx]

# Supplementary text

## **Supplementary Text 1**. Optical genome mapping.

Two whole blood samples and seventeen skin biopsies were obtained from the cohort. Skin biopsies were used to grow fibroblast cultures in Dulbecco’s Modified Eagle’s Medium supplemented with 10% fetal bovine serum and 1% penicillin/streptomycin (Thermo Fisher Scientific). Genomic ultra-high molecular weight DNA (UHMW) was isolated from the blood samples and fibroblast cell pellets with the "Bionano Prep SP-G2 Frozen Human Blood DNA Isolation Protocol" or the "Bionano Prep SP-G2 Frozen Cell Pellet DNA Isolation Protocol", depending on the available biomaterial, according to the manufacturer's instructions. In brief, after lysis of the surrounding tissue, the UHMW DNA was precipitated on a nanobind magnetic disk using isopropanol (≥99.5%) and washed. Afterward, the UHMW DNA was eluted, homogenized, and quantified using Qubit broad-range double-stranded DNA assay kits (Thermo Fisher Scientific). DNA labeling was conducted according to the "Bionano Prep DLS-G2 Protocol". 750 ng of the UHMW DNA were incubated with the DLE-1 enzyme and DL-Green fluorophores, resulting in covalently attached labels at specific sequence motifs. After the proteolytic digest of DLG-1, removal of the remaining fluorophores, and another quantification step, the labeled DNA molecules were loaded on a Saphyr flowcell chip and imaged on the Bionano Saphyr. Following the optical imaging, the generated optical maps were processed with the *de novo* pipeline included in the Bionano Solve software (v3.8). During this process, the images are aligned against each other, assembled, and compared to the GRCh38 reference genome to identify SVs. A single sample that carried a known 7 Mb inversion affecting *PRKN* was aligned to the T2T-CHM13v2.0 genome reference. The subsequent results of the *de novo* pipeline were analyzed via the Bionano Access software (v1.8), and the identified SVs and CNVs were exported as VCF and SMAP files containing the variants.

## **Supplementary Text 2**. Nanopore long-read sequencing

LRS was performed at two different facilities using UHMW DNA extracted for OGM: externally at the NGS Competence Center Tübingen (NCCT) (*N*=7) and internally at the Institute of Neurogenetics (ING) Lübeck (*N*=12). At the NCCT, the SQK-LSK109 (ONT) library kit and R9.4.1 flow cells (FLO-PRO002) were used for library preparation and sequencing on an ONT PromethION device. Pre-treatment with T7 endonuclease was performed prior to library preparation and flow cell loading according to the standard SQK-LSK109 protocol. The base-calling was performed using Dorado v0.6.3. (basecall server 7.4.12). At the ING, UHMW DNA was sheared with the Megaruptor 3 (Diagenode), and subsequent size selection was performed using the BluePippin (Sage Science). The Qubit fluorometer (Thermo Fisher Scientific), Femto Pulse system, and Tapestation 4200 (Agilent Technologies) were used to quantify the DNA and monitor the size range after shearing and size selection^26^. Library preparation and sequencing were conducted using the manufacturer's SQK-LSK114 protocol for genomic DNA, R10.4.1 flow cells (FLO-PRO114M), and the PromethION device from ONT. High accuracy base-calling (HAC) was performed using Dorado v0.6.3 in MinKNOW v24.06.10, generating output files (.fastq) for in-house analysis.

## **Supplementary Text 3**. PacBio long-read sequencing validations

PacBio LRS was additionally performed on a subset of three samples (L-3049, L-8302, 3049-10) to validate large outlying SVs with an orthogonal method. Genomic DNA was sheared with the Megaruptor 3 (Diagenode), and the Qubit fluorometer (Thermo Fisher Scientific), the Femto Pulse system, and the Tapestation 4200 (Agilent Technologies) were used to quantify the DNA and monitor the size range after shearing. The SMRTbell prep kit 3.0 (PacBio) was used for Library preparation, and the Vega polymerase kit (103-426-500) was used for sequencing on the Vega benchtop system (PacBio). The obtained reads were aligned to the GRCh38 reference genome using v2.28 of minimap2^27^, and the resulting BAM file was manually inspected in the Integrative Genomics Viewer (IGV).

# Supplementary figures

**Supplementary Figure 1: Variant filter steps.** High-quality SVs and CNVs identified with OGM and ONT were filtered based on allele frequency and position within known genes associated with movement disorders. Variants were considered high-quality with a Bionano confidence score greater than 0.99 (corresponding to a Phred quality score of 20). All ONT variants passed the quality criteria (FILTER=PASS) and exhibited a Phred quality score of greater than 20. Variants with an allele frequency of ≤0.01 in the internal Bionano control database (OGM) or the gnomAD 4.1 SV database (ONT) were considered rare. OFP: Overlap fraction product. Created in BioRender. Fienemann, A. (2025) BioRender.com/m8t8xni.

**Supplementary Figure 2: Coding Variants**. High-quality SVs and CNVs identified with OGM and ONT were filtered based on overlap with coding sequences/exons, as per the AnnotSV annotations. The resulting coding OGM variants were compared to ONT variants ≥500 bp in four categories: 1. small deletions ≤50 kb, 2. small insertions ≤50 kb, 3. intermediate/large deletions ≥50 kb, 4. intermediate/large insertions ≥50 kb. A fixed interval (±6 kb and 15 kb) regarding the start and end of the variants was applied based on the median 95% confidence interval of the OGM calls. The resulting subsets were also filtered based on overlap with coding sequences/exons. CDS: coding sequence, DEL: deletion, INS: insertions. Created in BioRender. Fienemann, A. (2025) BioRender.com/vh2s31v.

**Supplementary Figure 3: ECDF comparison.** Empirical cumulative distribution functions (ECDFs) illustrate the composition of insertion and deletion lengths detected by OGM and ONT. All OGM ECDFs are shifted to a longer variant length compared to ONT. **A**. Comparison of all deletions over the size of 500 bp (resolution of OGM). **B**. Comparison of all insertions over the size of 500 bp. **C**. Comparison of all deletions ≤50 kb. **D**. Comparison of all deletions >50 kb. INS: insertion, DEL: deletion.

**Supplementary Figure 4: Concordance of MDS variants between ONT and OGM**. These Venn diagrams show the simplified overlap of deletions (**A**) and insertions (**B**) between SVs and CNVs (≥500 bp) overlapping with known genes associated with movement disorders identified by ONT and OGM. Due to the difference in methodology, the SVs in the overlap fraction are not exact matches. For the overlap, a fixed interval (±7 kb) regarding the start and end of the variants was applied based on the median 95% confidence interval of all OGM calls affecting MDS genes.

**Supplementary Figure 5: IGV snapshot of a *ATXN2* deletion.** The figure shows a snapshot from the Integrative Genomics Viewer (IGV) displaying ONT reads mapped to Chromosome 12 of the GRCh38 reference genome. A heterozygous 1.4 kb deletion was identified at the position chr12:111593192—111594604.

**Supplementary Figure 6: IGV snapshot of a *PNKD* deletion.** The figure shows a snapshot from the Integrative Genomics Viewer (IGV) displaying ONT reads mapped to Chromosome 2 of the GRCh38 reference genome. A heterozygous 2.6 kb intronic deletion was identified at the position chr2:218330537—218333189.

 **Supplementary Figure 7: IGV snapshot of a *SUCLA2* deletion.** The figure shows a snapshot from the Integrative Genomics Viewer (IGV) displaying ONT reads mapped to Chromosome 13 of the GRCh38 reference genome. A heterozygous 1.7 kb intronic deletion was identified at the position chr13:47968800—47970573.

**Supplementary Figure 8: Bionano Access snapshot of a 98 kb deletion spanning *PEX7*.** The figure shows a 98 kb deletion spanning most of the *PEX7* gene. Annotated in BioRender. Fienemann, A. (2025) BioRender.com/6fgj5cm.

**Supplementary Figure 9: IGV snapshot of a 98kb deletion spanning *PEX7*.** The figure shows a snapshot from the Integrative Genomics Viewer (IGV) displaying ONT reads mapped to Chromosome 6 of the GRCh38 reference genome. A heterozygous copy number reduction affecting *PEX7* (red bar) can be identified visually, indicating a deletion; however, no variant was called with the SV-caller Sniffles2 (v2.3.3).

**Supplementary Figure 10: Samplot snapshot of a 98 kb deletion affecting *PEX7*.** The deletion spanning the *PEX7* gene can be detected using v2.6.2 of the SV-caller Sniffles2. The visualization tool Samplot was used (Belyeu et al., 2021).
